# Serum SARS-COV-2 Nucleocapsid Protein: A Sensitivity and Specificity Early Diagnostic Marker for SARS-COV-2 Infection

**DOI:** 10.3389/fcimb.2020.00470

**Published:** 2020-09-04

**Authors:** Tao Li, Li Wang, Huihui Wang, Xuemei Li, Shubing Zhang, Yuanhong Xu, Wei Wei

**Affiliations:** ^1^Department of Clinical Laboratory, The First Affiliated Hospital of Anhui Medical University, Hefei, China; ^2^Key Laboratory of Anti-inflammatory and Immune Medicine, Ministry of Education, Anhui Collaborative Innovation Center of Anti-inflammatory and Immune Medicine, Anhui Anti-inflammatory and Immune Medicine Innovation Team, Institute of Clinical Pharmacology, Anhui Medical University, Hefei, China; ^3^Biohit Healthcare (Hefei) Co., Ltd, Hefei, China

**Keywords:** SARS-CoV-2, COVID-19, nucleocapsid protein, colloidal gold immunochromatography, diagnostic value

## Abstract

**Objective:** To explore the diagnostic value of serum severe acute respiratory syndrome coronavirus 2 (SARS-CoV-2) nucleocapsid (N) protein assay in the early stages of SARS-COV-2 infection.

**Methods:** Serum N protein level in SARS-COV-2 infected patients and non-SARS-COV-2 infected population was measured by enzyme-linked immunosorbent assay (ELISA) double antibody sandwich assay. Colloidal gold immunochromatography assay was used to detect serum N protein antibodies in the above populations.

**Results:** Fifty cases of SARS-CoV-2 nucleic acid-positive and SARS-CoV-2 antibody-negative patients had a serum N protein positivity rate of 76%. Thirty-seven patients who were positive for serum SARS-CoV-2 antibody after infection had a serum SARS-CoV-2 N protein positivity rate of 2.7%. Serum N protein test results of 633 non-SARS-COV-2 infected patients, including pregnant women, patients with other respiratory infections, and individuals with increased rheumatoid factor were all negative, with serum N protein concentration <10.00 pg/mL at 100% specificity. Using SPSS 19.0 to calculate the receiver operating characteristic curve, the area under the curve was determined to be 0.9756 (95% confidence interval 0.9485–1.000, *p* < 0.0001), and sensitivity and specificity were 92% (95% confidence interval 81.16–96.85%) and 96.84% (95% confidence interval 95.17–97.15%), respectively. The best CUT-OFF value was 1.850 pg/mL.

**Conclusion:** The measurement of serum SARS-COV-2 N protein has a high diagnostic value for infected patients before the antibody appears and shortens the window period of serological diagnosis. It is recommended that the manufacturer establish two different CUT-OFF values according to the purpose of the application. One CUT-OFF value is used for the diagnosis of clinical SARS-COV-2 infection, and the other is used to screen out as many suspected cases as possible.

## Introduction

As of July 10, 2020, there were more than 12.2 million people infected with severe acute respiratory syndrome coronavirus 2 (SARS-CoV-2) and more than 554,291 deaths (Johns Hopkins University CSSE, 2020; https://gisanddata.Maps.Arcgis.Com/apps/opsdashboard/index.Html#/bda7594740fd40299423467b48e9ecf6), which has posed a serious threat to the health and economic life of people globally. With the joint efforts of scientists worldwide, a variety of diagnostic reagents have been developed to provide support for the clinical diagnosis of SARS-COV-2 (Chan et al., 2020; Corman et al., 2020; Konrad et al., 2020; Reusken et al., 2020). At present, the diagnosis of SARS-COV-2 infection is mainly based on pharyngeal swab or sputum nucleic acid detection, and specific serum antibody detection is used as an auxiliary marker (To et al., 2020; http://www.nhc.gov.cn/yzygj/s7653p/202003/46c9294a7dfe4cef80dc7f5912eb1989.shtml). Nucleic acid testing is greatly affected by specimen collection, transportation, and other stages. The number of inconsistent positive and negative results is high, and the overall rate of positivity is not high (Abduljalil, 2020; Cheng et al., 2020; Xie et al., 2020), which causes great confusion during clinical diagnosis. The test for specific antibodies against SARS-COV-2 in the serum will appear positive only about 7 days after infection or later in severe coronavirus disease 2019 (COVID-19) (Qu et al., 2020; Zainol Rashid et al., 2020). It is difficult to detect the infection at an early stage, making it difficult to block the source of infection, and this increases the difficulty of preventing and controlling the spread of SARS-COV-2 infection. Given this situation, this study analyzed the positivity rate of serum N protein before the generation of serum antibodies in patients infected with SARS-COV-2, providing new diagnostic indicators for the early detection of SARS-COV-2 infection.

## Materials and Methods

### Specimen Source

#### COVID-19 Patients

Forty COVID-19 patients were enrolled from the First Affiliated Hospital of Anhui Medical University, and 30 COVID-19 patients were enrolled from the Anhui Provincial Center for Disease Prevention and Control. All COVID-19 patients were diagnosed based on the results of nucleic acid Reverse Transcription-Polymerase Chain Reaction (RT-PCR) test, as well as pathological changes observed in computed tomography (CT) images, according to the seventh edition of the pneumonia diagnosis and treatment program for the novel Coronavirus infection reported by the (National Health Commission of the People's Republic of China, 2020; http://www.Q13nhc.gov.cn/yzygj/s7653p/202003/46c9294a7dfe4cef80dc7f5912eb1989.shtml). Among the 40 COVID-19 patients enrolled from the First Affiliated Hospital of Anhui Medical University, 20 cases were negative for serum SARS-COV-2 N protein antibody on the first day of admission; later, 37 cases tested positive for serum SARS-COV-2 N protein antibody within 9 days of admission. We categorized the 70 COVID-19 patients into a SARS-COV-2 N protein antibody-positive group and a SARS-COV-2 N protein antibody-negative group. Thus, 50 samples from patients with positive pharyngeal swab or sputum SARS-COV-2 nucleic acid and negative serum SARS-COV-2 N protein antibody test results were collected from the First Affiliated Hospital of Anhui Medical University (20 cases) and the Anhui Provincial Center for Disease Prevention and Control (30 cases). Thirty-seven samples from patients with positive pharyngeal swab or sputum SARS-COV-2 nucleic acid and positive serum SARS-COV-2 N protein antibody test results were collected from the First Affiliated Hospital of Anhui Medical University.

#### Non-COVID-19 Patients

Six hundred thirty-three samples with negative pharyngeal swab or sputum SARS-COV-2 nucleic acid result and negative serum N protein antibody test results were obtained from the First Affiliated Hospital of Anhui Medical University, including 100 samples from pregnant women (serum of pregnant women at 15–30 weeks), 369 serum samples from patients with other respiratory infections (non-COVID-19 patients with respiratory symptoms), 119 serum samples from individuals with increased rheumatoid factor (more than one time above the upper limit of the reference value; reference value: 0–14 IU/ml), and 45 hemolytic samples. All samples were tested for SARS-CoV-2 N protein by ELISA double antibody sandwich assay.

### Reagents

#### The SARS-COV-2 Antigen Quantitative Detection Kit (ELISA) (Lot Number 20200508) Was Developed by BIOHIT Healthcare (Hefei) Co., Ltd.

##### Principle

The double antibody sandwich method was used to detect SARS-CoV-2 N protein in human serum. SARS-COV-2 N protein antibodies were pre-coated with an enzyme-labeled microplate. After the addition of the serum samples, samples containing SARS-COV-2 N protein could bind to the envelope plate. The antigen-antibody complexes then combined with the added biotin-labeled SARS-COV-2 N protein antibodies to form a sandwich complex of solid-phase antibody-antigen-biotinylated antibodies. Next, horseradish peroxidase (HRP)-labeled chain avidin (SA) was added, which binds to the biotinylated antibodies, followed by the addition of the HRP substrate chromogenic solution for color development.

##### Results interpretation

The positive interpretation value (cut-off value) is 10.00 pg/mL. If the concentration in the specimen to be tested was <10.00 pg/mL, the specimen was interpreted to be negative for SARS-COV-2 N protein. If the concentration in the specimen to be tested was ≥ 10.00 pg/mL, then the specimen was interpreted as positive for SARS-COV-2 N protein.

#### The SARS-COV-2 IgM/IgG Antibody Detection Kit (Colloidal Gold Method) (Batch Number SA200301) Was Developed by BIOHIT Healthcare (Hefei) Co., Ltd.

##### Principle

Colloidal gold immunochromatography was used to detect anti-SARS-CoV-2 IgM/IgG N protein antibody in human serum. The SARS-CoV-2 IgM/IgG was detected using the SARS-CoV-2 recombinant N-protein antigen and mouse anti-human IgM/IgG antibody. SARS-CoV-2 IgM/IgG in the sample reacted with SARS-CoV-2 recombinant N-protein antigen bound to gold particles. This complex migrated along the membrane and reached the IgM/IgG test line (T), which contains mouse anti-human IgM/IgG antibody against SARS-CoV-2 IgM/IgG complex. The sample diluent was supplied for the lateral chromatography process to provide a suitable environment for the reaction of antigen and antibody.

##### Results interpretation

If the quality control line C developed color, and the test line did not develop color, it was interpreted as a negative result. If the quality control line C developed color, and the test line also developed color, it was determined as a positive result. If the quality control line C did not develop color, the result was considered invalid, and the sample needs to be re-tested.

### Precision of the SARS-COV-2 Antigen Quantitative Detection Kit (ELISA)

The selected test values were 6.5, 43.6, and 164.8 pg/mL, which were called S1, S2, and S3, respectively.

Within-day precision: each specimen was tested 20 times a day (10 times in the morning, and 10 times in the afternoon). The mean value, standard deviation (SD), and coefficient of variation (CV) of the results were calculated for 20 times.

Day to day precision: each specimen was tested once a day for 20 consecutive days, and the mean value, SD, and CV of the results of 20 tests were calculated.

### Statistical Analysis

The data in this study are mainly descriptive. The proportions of specimens between groups were expressed as percentages, and the clinical efficacy was expressed with sensitivity and specificity. ROC curves were drawn using SPSS 19.0.

## Results

### The Positivity Rate of Serum N Protein When Pharyngeal Swab or Sputum Nucleic Acid Results Were Positive and Serum Antibody Results Were Negative for SARS-COV-2

Fifty cases of SARS-COV-2 nucleic acid-positive and antibody-negative patients had serum N protein positivity rate of 76%, including 2% with a concentration of 10.00–49.99 pg/mL, 8% with a concentration of 50.00–99.99 pg/mL, 22% with a concentration of 100.00–299.99 pg/mL, and 44% with a concentration ≥ 300.00 pg/mL. The negativity rate of serum N protein was 24%, including 12% with a concentration of 0.00–1.99 pg/mL, 10% with a concentration of 2.00–4.99 pg/mL, and 2% with a concentration of 5.00–9.99 pg/mL (the results are shown in Table 1).

**Table 1:** Serum N protein composition ratio among nucleic acid positive and antibody negative patients.

**Detection marker**	**N Protein conc**.	**Percentage**
Nucleic acid positive + antibody Negative +N Protein positive	**≥10.00 pg/mL**	**76% (38/50)**
	*10.00–49.99 pg/mL*	*2%* (*1/50)*
	*50.00–99.99 pg/mL*	*8%* (*4/50)*
	*100.00–299.99 pg/mL*	*22%* (*11/50)*
	*≥300.00 pg/mL*	*44%* (*22/50)*
Nucleic acid positive + antibody Negative +N Protein negative	** <10.00 pg/mL**	**24% (12/50)**
	*0.00–1.99 pg/mL*	*12%* (*6/50)*
	*2.00–4.99 pg/mL*	*10%* (*5/50)*
	*5.00–9.99 pg/mL*	*2%* (*1/50)*

*The meaning of the bold values provided is the total number. The meaning of the italics values provided is the number of people with different N protein concentration detected values*.

### The Positivity Rate of Serum N Protein When Pharyngeal Swab or Sputum Nucleic Acid Results Were Positive and Serum Antibody Results Were Positive for SARS-COV-2

Thirty-seven samples from patients positive for serum antibody after infection showed serum N protein positivity rate of 2.7%, of which 2.7% had a concentration of 10.00–49.99 pg/mL and 0% had concentrations of 50.00–99.99, 100.00–299.99, and ≥300.00 pg/mL. The negativity rate of N protein was 97.3%, of which 73.0% had a concentration of 0.00–1.99 pg/mL, 13.5% had a concentration of 2.00–4.99 pg/mL, and 10.8% had a concentration of 5.00–9.99 pg/mL (The results are shown in Table 2).

**Table 2:** Serum N protein composition ratio among nucleic acid positive and antibody positive patients.

**Detection marker**	**N Protein conc**.	**Percentage**
Nucleic acid positive +antibody Positive +N Protein positive	**≥10.00 pg/mL**	**2.7% (1/37)**
	*10.00–49.99 pg/mL*	*2.7%* (*1/37)*
	*50.00–99.99 pg/mL*	*0%* (*0/37)*
	*100.00–299.99 pg/mL*	*0%* (*0/37)*
	*≥300.00 pg/mL*	*0%* (*0/37)*
Nucleic acid positive + antibody Positive +N Protein negative	** <10.00 pg/mL**	**97.3% (36/37)**
	*0.00–1.99 pg/mL*	*73.0%* (*27/37)*
	*2.00–4.99 pg/mL*	*13.5%* (*5/37)*
	*5.00–9.99 pg/mL*	*10.8%* (*4/37)*

*The meaning of the bold values provided is the total number. The meaning of the italics values provided is the number of people with different N protein concentration detected values*.

### Analysis of Antibody Titer and CT Results of Four Patients Whose N Proteins Were Consistently Negative

**Figure 1:**
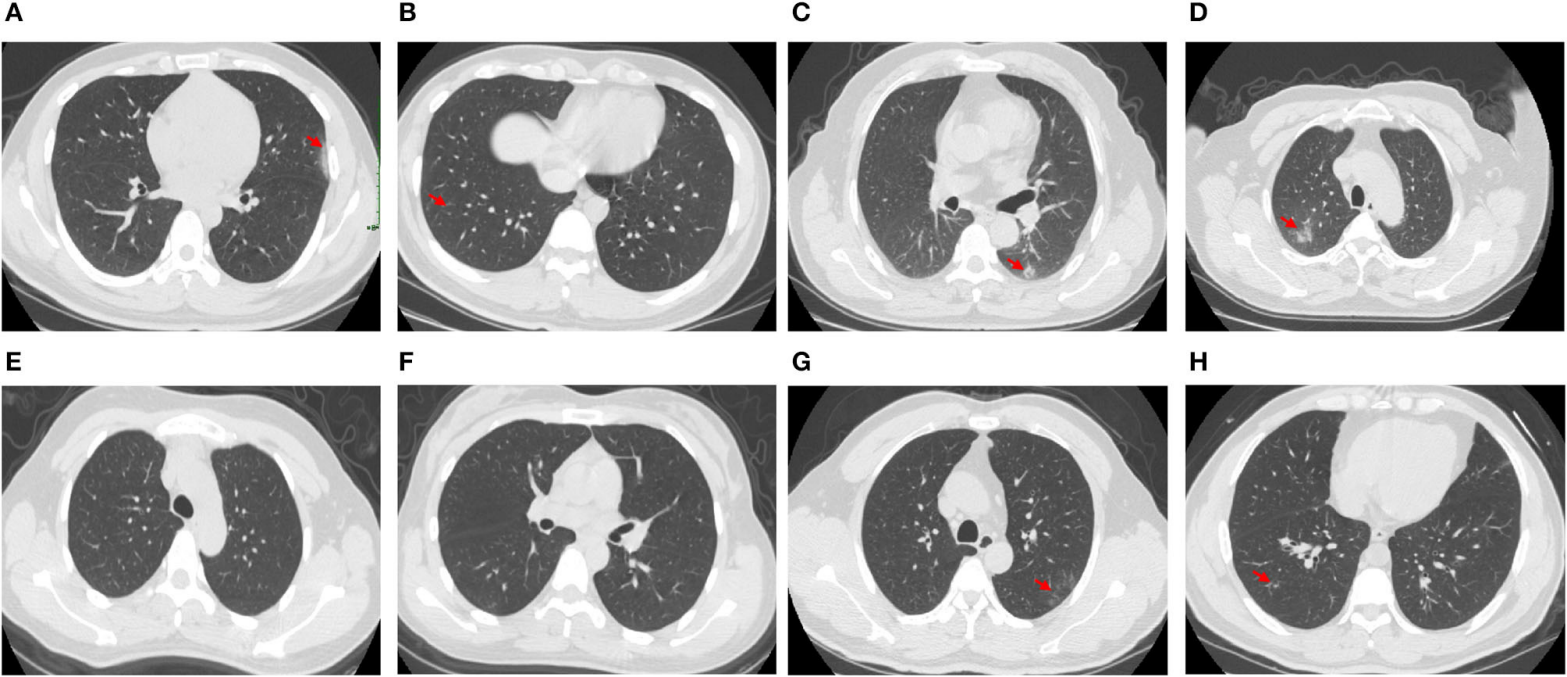
CT images of 4 patients whose N protein is consistently negative, **(A,B)**, CT images of Patient 3 1–3 days after admission; **(C,D)**, CT images of Patient 32 1–3 days after admission; **(E,F)**, CT images of Patient 33 1–3 days after admission; **(G,H)**, CT images of Patient 40 1–3 days after admission.

Among the COVID-19 patients whose N proteins were consistently negative, the serum antibody and CT results of four from our hospital were tracked. The serum antibody was negative or in low titer for more than 9 days after the infected patients were admitted to the hospital. CT imaging showed that the patient's lungs were mildly inflamed or not inflamed at all (The results are shown in Table 3 and Figure 1).

**Table 3:** Antibody titer and CT results of 4 patients whose N protein is consistently negative.

**Patient ID**	**Marker**	**1–3 days after admission**	**4–6 days after admission**	**7–9 days after admission**	**>9 days after admission**
3	Antibody titer	Negative	Negative	1:1*	1:1*
	CT imaging	A little inflammation under the pleura of the right lower lobe and under pleura of the left upper lobe	A little inflammation under the pleura of the right lower lobe and in the upper left lung	A little inflammation of the left lung	A little inflammation of the left lung
32	Antibody titer	Negative	Negative	1:1*	1:1*
	CT imaging	A little inflammation in the right upper lobe and left lower lobe	A little inflammation in the right upper lobe and left lower lobe, improved from previous exam	A little inflammation in the right upper lobe and left lower lobe, improved from previous exam	A little inflammation in the right upper lobe and left lower lobe, improved from previous exam
33	Antibody titer	1:1*	1:1*	1:1*	1:1*
	CT imaging	No obvious lesions in both lungs	No obvious lesions in both lungs	No obvious lesions in both lungs	No obvious lesions in both lungs
40	Antibody titer	Negative	Negative	Negative	Negative
	CT imaging	A little inflammation of the left upper lobe	A little inflammation of the left upper lobe, improved from the previous exam	A little inflammation of the left upper lobe, improved from the previous exam	A little inflammation of the left upper lobe, improved from the previous exam

1:1 *means when the serum is tested as undiluted, it showed weak positive results. Further dilution would result in negative reading.

### Specificity of SARS-COV-2 Serum N Protein Assay at the CUT-OFF Value Recommended by the Manufacturer

Serum N protein test results of 633 patients with non-SARS-COV-2 infection showed a specificity of 100%, including serum samples from 100 pregnant women, 369 samples from patients with other respiratory infections, 119 serum samples from individuals with elevated rheumatoid factor, and 45 hemolytic samples (The results are shown in Table 4).

**Table 4:** Specificity of SARS-COV-2 serum N protein assay at the CUT-OFF value recommended by the manufacturer or calculated by using the ROC curve.

**Sample source**	**No. of sample**	**The CUT-OFF value recommended by the manufacturer**		**The CUT-OFF value calculated by using the ROC curve**	
		**Number of antigen positive samples**	**Specificity (%)**	**No. of N protein positive sample**	**Specificity (%)**
Serum samples of pregnant women	100	0	100	9	91.0
Serum samples with other respiratory infections	369	0	100	10	97.3
Serum samples with elevated rheumatoid factor	119	0	100	0	100
Hemolytic samples	45	0	100	1	97.8
Total	633	0	100	20	96.8

### Receiver Operator Characteristic Curve (ROC Curve)

**Figure 2:**
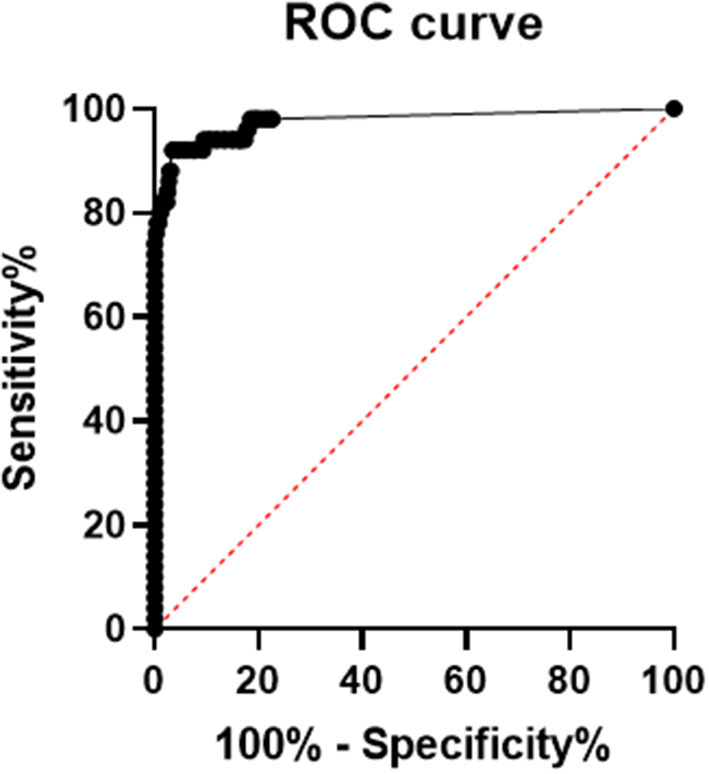
ROC curve of serum SARS-COV-2 N protein, area under the ROC curve is 0.9756, 95% confidence interval (CI) is 0.9485 to 1.000, *p*-value is lower 0.0001, sensitivity and specificity are 92% (95% CI 81.16 to 96.85%) and 96.84% (95% CI 95.17 to 97.15%), respectively. The best CUT-OFF value is 1.850 pg/mL.

Using SPSS 19.0 to calculate the receiver operating characteristic curve, the area under the curve was determined to be 0.9756 (95% confidence interval, 0.9485–1.000; *p* < 0.0001), and sensitivity and specificity were 92% (95% confidence interval, 81.16–96.85%) and 96.84% (95% confidence interval, 95.17–97.15%), respectively. The best CUT-OFF value was found to be 1.850 pg/mL (The results are shown in Figure 2). When calculating the CUT-OFF value using the ROC curve, the specificity of pregnancy population samples, other respiratory infection samples, elevated rheumatoid factor samples, and hemolytic samples were 91.0, 97.3, 100, and 97.8%, respectively (The results are shown in Table 4).

### Distribution of N Protein Concentration Result of Sample Data

**Figure 3:**
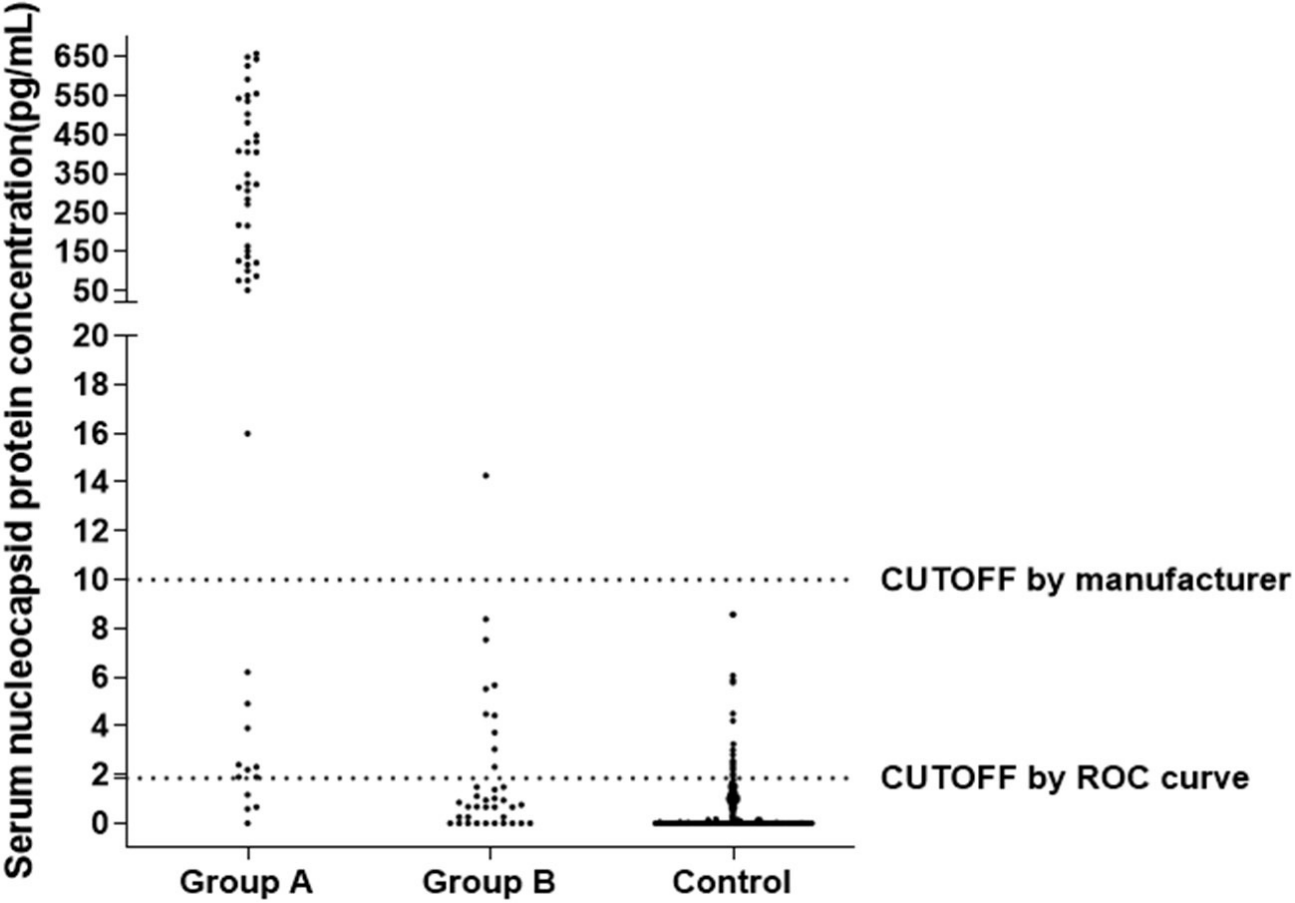
Distribution of N Protein concentration result of sample data using the CUT-OFF value equaling to 10, Group A (Nucleic Acid Positive + Antibody Negative, *N* = 50), Group B (Nucleic Acid Positive + Antibody Positive, *N* = 37), Control Group (non-SARS-COV-2 infection, *N* = 633).

Figure 3 shows the distribution of sample results using the CUT-OFF value of 10.00 pg/mL or 1.850 pg/mL. In Group A (Nucleic Acid Positive + Antibody Negative, *N* = 50), the distribution of samples was mostly above 10.00 pg/mL, with a large spread from zero. In Group B (Nucleic Acid Positive + Antibody Positive, *N* = 37) and the Control Group (non-SARS-COV-2 infection, *N* = 633), the distribution of samples was mostly below 10.00 pg/mL, with a cluster of samples around zero.

### Precision Analysis of the SARS-COV-2 Antigen Quantitative Detection Kit (ELISA)

Within-day precision and day-to-day precision of SARS-COV-2 antigen Quantitative detection Kit (ELISA) were analyzed by clinical specimens with low value (6.5 pg/mL), median value (43.6 pg/mL) and high value (164.8 pg/mL). The within-day precision was 8.65%, 6.51%, and 5.80%, and the day-to-day precision was 9.52, 7.47, and 6.23%, respectively (The results are shown in Table 5).

**Table 5:** Precision analysis of the SARS-COV-2 antigen quantitative detection kit (ELISA).

	**Within-day precision**			**Day to day precision**		
	**S1**	**S2**	**S3**	**S1**	**S2**	**S3**
Mean value(pg/mL)	6.49	41.15	163.10	6.51	41.13	166.33
SD(pg/mL)	0.56	2.68	9.46	0.62	3.07	10.36
CV(%)	8.65%	6.51%	5.80%	9.52%	7.47%	6.23%

## Discussion

Pulmonary inflammation is most common with SARS-CoV-2 infection, and the virus can also invade other tissues (Farkash et al., 2020; Yao et al., 2020). Some patients develop viremia. It was reported that SARS-CoV-2 RNA was detected in the blood among 15% of the 41 COVID-19 patients who were admitted to the hospital for the first time in Wuhan (Huang et al., 2020). The results of this study showed that 76.8% of patients diagnosed with SARS-CoV-2 infection were positive for N protein before the emergence of the N antibody (see Table 1). Further studies are needed to confirm whether infected patients have a higher incidence of viremia in the early stages or whether the over-expressed N protein of the lung virus spills into the blood.

Recently, many new detection technologies for SARS-COV-2 have been reported, such as luciferase IP system assay and lateral flow assays (Grant et al., 2020; Haljasmägi et al., 2020). Serum SARS-COV-2 antibody test has been widely recognized as serological evidence for the diagnosis of COVID-19 (Cai et al., 2020; Shen et al., 2020; Sun et al., 2020). However, due to the lag in the development of antibodies, the “window period” for clinical diagnosis is too long, and serum SARS-COV-2 antibody test is not suitable for the early diagnosis of SARS-COV-2 infection (Qu et al., 2020; Sun et al., 2020; Tripathi et al., 2020). Our research results showed that when the serum antibody is positive, the serum N protein positivity rate is only 2.7%, and the overall serum N protein concentration value is low (see Table 2), suggesting that the detection of serum antibody and N protein has good complementary effects for the diagnosis of SARS-COV-2 infection. The results in Table 3 show that four patients negative for serum N protein had low antibody titers, and their CT showed that the lesions in these patients were mild or inflammation was absent; whether this is related to viral load *in vivo* remains to be further studied.

The results in Tables 1, 4 show that using the CUT-OFF value recommended by the manufacturer, the specificity of serum N protein assay could reach 100%, but the sensitivity only reaches 76.8%. If the ROC curve is used to establish a personalized CUT-OFF value, its specificity decreases to 96.84%, but its sensitivity increases to 92%. Therefore, we suggest that laboratories should select appropriate CUT-OFF values according to the intended use. If a higher diagnostic specificity is intended, we recommend the use of CUT-OFF values recommended by the manufacturer. If finding as many infected people as possible and establishing the basis for controlling the source of infection is the intention, it is recommended to select the CUT-OFF value obtained from the ROC curve.

The results in Table 5 show that the within-day precision and day-to-day precision of the low-value specimens are both <10% and that the within-day precision and day-to-day precision of the medium and high-value specimens are both <8%. The repeatability thus meets clinical requirements.

The results of this study provide a laboratory basis for the detection of serum SARS-COV-2 N protein for the early diagnosis of infection. Compared to the detection of SARS-COV-2 RNA from pharyngeal swab or sputum, serum SARS-COV-2 N protein detection has obvious advantages in specimen collection and treatment. For example, N protein has better stability than RNA, which may effectively make up for missed diagnosis caused by RNA false-negative results due to various reasons. Although the number of cases in this study was small, our results are extremely encouraging, which brings a new thought to the current incomplete diagnosis of SARS-COV-2 infection. The combined detection of three markers, pharyngeal swab or sputum SARS-COV-2 RNA, serum N protein, and serum antibody, may be the direction for diagnosis of SARS-COV-2 infection in the future.

## Conclusion

1. The detection of SARS-COV-2 serum N protein has a high diagnostic value for infected patients before the appearance of antibodies and shortens the window of serological diagnosis.
2. According to the CUT-OFF value recommended by the manufacturer, the specificity of SARS-COV-2 serum N protein detection is 100%, and the sensitivity is 76.8% before the appearance of antibodies.
3. Based on the CUT-OFF value determined from the ROC curve, the specificity of the SARS-COV-2 serum N protein detection was 96.84%, and the sensitivity was 92% before the appearance of antibodies.
4. It is recommended that the manufacturer establish two different CUT-OFF values according to the purpose of the application. One CUT-OFF value is used for the diagnosis of clinical SARS-COV-2 infection, and the other is used to screen out as many suspected people as possible.

## Data Availability Statement

All datasets presented in this study are included in the article/supplementary material.

## Ethics Statement

This study was approved by the Ethics Committee of the First Affiliated Hospital of Anhui Medical University. The patients/participants provided their written informed consent to participate in this study.

## Author Contributions

TL and LW had the idea for and designed the study, had full access to all data in the study, take responsibility for the integrity of the data, and the accuracy of the data analysis. HW, XL, and SZ contributed to writing of the report. YX and WW contributed to the statistical analysis. All authors contributed to data acquisition, data analysis, or data interpretation, and reviewed and approved the final version.

## Conflict of Interest

LW was employed by the company Biohit Healthcare (Hefei) Co., Ltd. The remaining authors declare that the research was conducted in the absence of any commercial or financial relationships that could be construed as a potential conflict of interest.
